# Research Progress on the Pathological Mechanisms of Podocytes in Diabetic Nephropathy

**DOI:** 10.1155/2020/7504798

**Published:** 2020-07-08

**Authors:** Lili Zhang, Zhige Wen, Lin Han, Yujiao Zheng, Yu Wei, Xinmiao Wang, Qing Wang, Xinyi Fang, Linhua Zhao, Xiaolin Tong

**Affiliations:** ^1^Department of Endocrinology, Guang'anmen Hospital, China Academy of Chinese Medical Sciences, Beijing 100053, China; ^2^Graduate College, Beijing University of Traditional Chinese Medicine, Beijing 100029, China

## Abstract

Diabetic nephropathy (DN) is not only an important microvascular complication of diabetes but also the main cause of end-stage renal disease. Studies have shown that the occurrence and development of DN are closely related to morphological and functional changes in podocytes. A series of morphological changes after podocyte injury in DN mainly include podocyte hypertrophy, podocyte epithelial-mesenchymal transdifferentiation, podocyte detachment, and podocyte apoptosis; functional changes mainly involve podocyte autophagy. More and more studies have shown that multiple signaling pathways play important roles in the progression of podocyte injury in DN. Here, we review research progress on the pathological mechanism of morphological and functional changes in podocytes associated with DN, to provide a new target for delaying the occurrence and development of this disorder.

## 1. Introduction

Diabetic nephropathy (DN) is a common microvascular complication in diabetes, with a prevalence rate of 30–40% in patients with type 1 or type 2 diabetes [1]; DN also accounts for 30–47% of end-stage renal disease (ESRD). It is the main cause of death in diabetic patients and the main cause of renal failure in ESRD [2]. DN accounts for 54% of new ESRD [3], and about 30% of chronic dialysis patients [4, 5]. With the developing economy, change in diet, and decreasing physical activity, the incidence of DN is increasing. DN is a progressive process. The early clinical manifestations are glomerular hyperfiltration and increased urinary albumin excretion rate. The pathological features are glomerular basement membrane thickening, mesangial dilatation, and tuberous sclerosis [6, 7]. With the development of DN, the number of damaged glomeruli increases and the glomerular filtration rate decreases significantly. The clinical manifestations are massive proteinuria, and glomerular and tubulointerstitial fibrosis [8, 9]. More and more studies have shown that the occurrence and development of DN are closely related to podocyte injury [10]. Podocytes are a unique and highly differentiated terminal glomerular epithelial cell and are attached to the outside of the glomerular basement membrane (GBM) to form a glomerular filtration barrier together with endothelial cells and the GBM. Podocytes are an indispensable part of the glomerular filtration barrier. The morphological changes in podocytes after injury in DN include podocyte hypertrophy, podocyte epithelial-mesenchymal transdifferentiation (EMT), podocyte detachment, and podocyte apoptosis [11]. The main functional changes involve podocyte autophagy. This article reviews research progress on the pathological mechanisms related to the morphological and functional changes of podocytes in DN.

## 2. Functional Changes of Podocytes

### 2.1. Autophagy

Autophagy was first proposed by Belgian scientist Christian de Duff in 1963, after Ashford and Porter discovered the phenomenon of “self-eating” in cells in 1962 [12]. Subsequent studies focused on the regulatory mechanisms of autophagy and its effects on human health and disease. Autophagy is a highly conserved process of intracellular protein recycling, which involves transferring damaged proteins and organelles to lysosomes for degradation; autophagy serves to mediate the recycling of intracellular nutrients, the continuous renewal of organelles, and the maintenance of intracellular homeostasis [13]. According to the different types of degraded substrates, the function of autophagy in cells is mainly classified as selective or nonselective autophagy [14–16]; in a nutrient-deficient environment, the recycling of intracellular energy sources is termed nonselective autophagy [17], and the removal of cytotoxic proteins and damaged organelles under different emergency conditions is known as selective autophagy [18]. In addition, depending on the different ways in which intracellular substrates are transported to lysosomes, autophagy can be divided into three types: macroautophagy, microautophagy, and molecular chaperone-mediated autophagy [19]; macroautophagy is the most widely studied process at present [20] and is the focus of this review.

### 2.2. Podocyte Autophagy and DN

Autophagy is a defense mechanism that is essential for maintaining podocyte homeostasis [21]. One study found that under normal circumstances, podocytes maintain a high level of autophagy for a long time [22]. However, there is a downregulation of podocyte autophagy activity in DN [23]. Continuous high glucose (HG) in DN can inhibit the expression of autophagy-related proteins Beclin-1, Atgl2, and LC3-II, weaken podocyte autophagy, and prevent the timely removal of damaged proteins and cytotoxins produced by organelle accumulation, resulting in irreversible podocyte damage and dysfunction [24, 25]. Tagawa et al. [26] directly revealed the progress of podocyte autophagy in DN for the first time. Presently, it has been found that a variety of signal pathways are involved in the regulation of podocyte autophagy, among which DN is closely related to mammalian target of rapamycin (mTOR), AMP-activated protein kinase (AMPK), oxidative stress, NAD+-dependent histone deacetylase, silent information regulatory factor-1 (Sirt1) signal pathway, Atg12-ATG5 coupling system, and vascular endothelial growth factor (VEGF).

#### 2.2.1. mTOR Signaling Pathway

mTOR is an evolutionarily highly conserved serine/threonine-protein kinase, which plays a key role in regulating cell growth and proliferation. It is very important to inhibit autophagy [27–31]. mTOR exists widely in eukaryotes. In mammals, it combines with different proteins to form two complexes with different structures and functions, mTORC1 and mTORC2. mTORC1 is sensitive to rapamycin and is mainly involved in the regulation of cell growth and development, proliferation, apoptosis, metabolism, autophagy, and so on. Studies have shown that the pathogenesis of DN is related to the activity of the mTORC1 pathway [22]. In a HG environment, mTORC1 was activated and protective autophagy was inhibited. The expression of mTORC1 was found to be upregulated in all patients with DN. MTORC1 was highly activated after knockout of a podocyte-specific upstream inhibitor of mTOR gene tuberous sclerosis complex 1 (TSC1), which inhibited autophagosomes by activating UNC-51-like kinase (ULK1) activity, resulting in podocyte damage [28, 32, 33]. In addition, rapamycin increases the number of LC3-expressing podocytes, promotes podocyte autophagy, and ameliorates renal injury in diabetic mice [34]. It is suggested that mTORC1 activity plays a key regulatory role in DN podocyte injury. Recently, Liu et al. [35] research confirmed that rapamycin inhibits mTOR activity, thereby regulating the pathological autophagic process. These studies suggest that mTOR activation in podocytes is the key reason for the occurrence and development of DN.

#### 2.2.2. AMPK Signal Pathway

AMPK is a heterotrimeric protein composed of one catalytic subunit (*α* subunit) and two regulatory subunits (*β* and *γ* subunits) of a serine protein kinase [36]. It plays an important role in the cells and tissues of patients with DN and is also an important metabolic stress protein kinase. AMPK can be activated by an increase in Ca^2+^ concentration in the cytoplasm [37, 38] and by the stimulation of numerous hormones, adipokines, and cytokines. Additionally, a decrease in the ratio of intracellular AMP/ATP activates AMPK, as does nutrient starvation. Under HG conditions, the phosphorylation level of AMPK is decreased and its activity is inhibited. AMPK could inhibit mTORC1 activity and induce autophagy through TSC1/2-Rheb signaling pathways and/or phosphorus acidification of raptor-related regulatory protein [31]. Additionally, AMPK directly mediates the phosphorylation of Ulkl/2 and induction of autophagy [39]. Other studies have shown that resveratrol, an AMPK activator, can reduce the early renal injury of DN by restoring the activity of AMPK in streptozotocin-induced diabetic rats [40]. Jin et al. [41] have shown that berberine can reduce HG-induced mouse podocyte autophagy by enhancing the activity of AMPK.

#### 2.2.3. Oxidative Stress

In 2001, Brownlee proposed that oxidative stress is commonly involved in the pathogenesis of diabetes and its complications [42]. Under HG conditions, cells produce a large number of advanced glycation end products (AGEs), which accumulate in the cells. In the process of AGE production, mitochondria release a large amount of reactive oxygen species (ROS), and excess ROS disturbs the balance of the oxidation and antioxidant systems, resulting in cell damage. Specifically, AGEs produced by cells stimulated by HG can upregulate the expression of the angiotensin II (AngII) receptor [43]. An in vitro podocyte experiment also showed that the expression of AngII increased under HG conditions [44]. Stimulated by AngII, podocytes increase ROS production and autophagy activation [45]. A study by Ma et al. [46] found that exposure to HG for 24 hours activated podocyte autophagy, through an upregulation of ROS production, and they also found that HG induced the generation of ROS by podocytes in a time-dependent manner. Wang et al. [47] found that the Rho/ROCK signal pathway may be activated, and Drp1 at serine 600, as a substrate of the Rho signal pathway, can initiate mitochondrial ROS under HG conditions. In addition, under the stimulation of HG, excess ROS can activate the intracellular AMPK signal pathway in the kidney [48]. Excess ROS can activate PKR-like kinase (PERK) to oxidize Atg4 protease by eIF2a phosphorylation, promote LC3 proteolysis, and prevent mTOR activation [49]. However, excess ROS will destroy the mitochondrial membrane, and the release of ROS into the cytoplasm may damage other organelles. As the function of autophagy targeting and degrading damaged organelles is selective, so the increase in ROS is limited [50]. Fang et al. [21] found that chronic exposure to HG conditions leads to autophagy insufficiency and subsequently causes lysosomal dysfunction and podocyte apoptosis, finally resulting in DN. Therefore, reduction of ROS generation is a potential therapeutic approach for preventing the development of DN.

#### 2.2.4. Sirt1 Signal Pathway

Sirt1 is a highly conserved NAD+-dependent III histone deacetylase, which exists widely in embryos and human tissues. At present, autophagy mediated by Sirt1 has attracted much attention. Sirt1 plays an important role in cells by deacetylating autophagy-related gene (Atg) products, such as Atg5, Atg7, Atg8, and forkhead protein transcription factor 3a [51], activating autophagy body formation and promoting autophagy [52]. Among them, Sirt1 plays an important role in the regulation of autophagy through the deacetylation of transcription factor fork frame O3 (FoxO3). Sirt1 is closely related to the occurrence and development of DN. Under the condition of HG, the expression of Sirt1 is inhibited, which increases oxidative stress and triggers apoptosis. For example, studies in db/db mice and streptozotocin-induced mice have found that the expression of Sirt1 is significantly downregulated before the occurrence of proteinuria [53]. Studies have shown that in proximal tubule cells, Sirt1 decreases albuminuria in diabetes mellitus through maintaining nicotinamide mononucleotide concentrations around glomeruli and controlling podocyte function [53, 54]. The above research shows that Sirt1 has a regulatory effect on autophagy.

#### 2.2.5. Atg12-Atg5 Conjugated System

Autophagy originates from the endoplasmic reticulum, and its formation includes initiation, nucleation, prolongation, and closure. Each step is strictly regulated by Atg coding products. The Atg12-Atg5 conjugated system is a class of Atg proteins closely related to podocyte autophagy. In vitro experiments showed that the autophagy activity of podocytes exposed to HG was significantly decreased, which was characterized by a significant decrease in the expression of autophagy-associated protein Atg12-Atg5 [22]. The activation of the Atg12-Atg5 conjugated system promotes the production of autophagosomes and activates podocyte autophagy. Studies have shown [55] that in a HG environment, the *β*-suppressor protein in the intracellular signal protein of G protein-coupled receptors inhibits podocyte autophagy by downregulating the Atg12-Atg5 conjugated system, which leads to the occurrence and development of DN.

#### 2.2.6. VEGF

**Figure 1:**
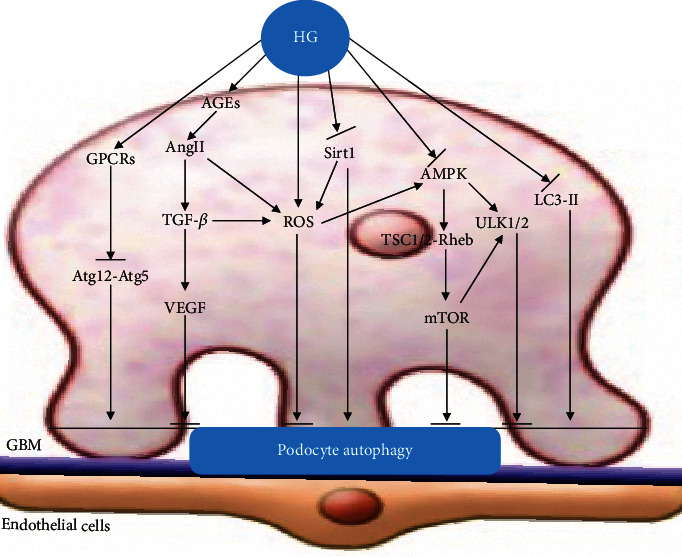
Podocyte autophagy. This figure illustrates the signal pathways of podocyte dysfunction in DN. HG inhibits podocyte autophagy by activating mTOR, ROS, and VEGF signaling pathways and inhibiting Sirt1, AMPK, LC3-II, and Atg12-Atg5 signaling pathways.

VEGF, synthesized by podocytes, is the promoter of angiogenesis and plays a key role in the maintenance of endothelial cell function [56], is the main pathogenic medium and important marker of DN, and participates in the occurrence and development of DN. In a HG environment, many factors activate transforming growth factor- (TGF-) *β*1. Through the binding of type I and type II receptors on the podocyte membrane, downstream Smad2 or Smad3 phosphorylation is induced, and the complex formed by binding with Smad4 and translocates to the nucleus, thus stimulating the secretion of VEGF [57]. The increase in VEGF is related to the increase of glomerular permeability [58]. In addition, VEGF, ROS, and AngII can stimulate TGF-*β*, which leads to renal hypertrophy, accumulation of mesangial extracellular matrix, and changes in podocyte morphology and function through Smad signal transduction. Studies have shown that HG increases VEGF levels by downregulating autophagy activity [32] Miaomiao et al. [59] found that diabetes caused podocyte foot process effacement and a significant upregulation of VEGF. In vitro, HG induced VEGF and reduced podocyte viability. After treatment with rapamycin in podocytes, an autophagy inducer, VEGF activation was significantly abrogated and podocyte injury was ameliorated. These studies show the vital role of autophagy in the regulation of VEGF, which serves as a protective mechanism against HG-induced podocyte injury (Figure 1).

## 3. Morphological Changes of Podocytes

Podocytes play an important role in the development of DN. A series of signal transduction pathways and changes in the renal microenvironment, involving related proteins and growth factors, lead to morphological cell damage and DN progression. Podocytes in DN are prone to a series of morphological changes after injury, including podocyte hypertrophy, podocyte EMT, podocyte detachment, and podocyte apoptosis [11].

### 3.1. Podocyte Hypertrophy

#### 3.1.1. mTOR Signal Pathway

mTOR signaling mainly comprises mTOR complex 1(mTORC1) and mTOR complex 2 (mTORC2). Several studies have suggested that mTORC1 is closely associated with the activation of podocyte hypertrophy, which is induced by HG [32]. Research by Herbach et al. [60] indicates that podocyte hypertrophy is directly linked to hyperglycemia. Gödel et al. [61] found that tightly balanced mTOR activity in podocyte homeostasis is required and suggest that mTOR inhibition can protect podocytes and prevent progressive DN. Kim et al. [62] found that translationally controlled tumour protein (TCTP), a mediator of cell growth, was overexpressed in the glomeruli of diabetic mice and gave rise to podocyte hypertrophy. Studies showed that TCTP could activate the mTORC1 signaling pathway and promote high expression of cyclin-dependent kinase inhibitors (CKIs), which caused podocyte cycle arrest and hypertrophy. In contrast, overexpression of mTORC1 and CKIs could be inhibited by TCTP knockout, to make the podocyte bodies smaller. Additionally, in vitro experiments indicated that a TCTP inhibitor could downregulate the expression of CKIs, ameliorating podocyte hypertrophy caused by HG. In addition, Das et al. found that Akt2 causes TGF-*β*-induced deptor downregulation facilitating mTOR to drive podocyte hypertrophy [63].

#### 3.1.2. TGF-*β* Signal Pathway

TGF-*β* is a multifunctional cytokine that mediates multiple signal pathways leading to podocyte hypertrophy in the pathogenesis of DN. It has been shown that exposure of differentiated podocytes to hyperglycemic conditions in vitro results in the upregulation of TGF-*β* expression [64, 65]. HG also augments the response of the podocyte to ambient levels of TGF-*β* [64]. Das et al. [66] believe that the production of proteinuria in patients with DN is related to TGF-*β*1, which can induce podocyte apoptosis, and found that TGF-*β*1 can induce the increase of podocyte mitochondrial NADPH oxidase, inhibiting podocyte mitochondrial function. From this, it can be inferred that TGF-*β* overexpression can induce podocyte hypertrophy and cause shape deformity. A HG environment increases the phosphorylation of Akt2 in glomerular podocytes, and TGF-*β* increases the phosphorylation of Akt2 and upregulates mTOR by stimulating PI3 kinase. Importantly, inhibition of Akt2 blocked TGF-*β*-induced podocyte hypertrophy [63]. In addition, TGF-*β* possesses an activation effect on the ERK pathway [67], and ERK1/2 activation is related to glomerular podocyte hypertrophy [68].

#### 3.1.3. AngII Signaling Pathway

HG induces the activation of the local renin-angiotensin system (RAS), which leads to an increase in AngII. AngII is an important mediator of DN and plays a key role in its occurrence and development [57, 69]. HG reportedly caused cultured podocytes to become hypertrophic in vitro, possibly through AngII [70]. Romero et al. [71] observed that PTHrP plays a key role in the mechanisms of HG-induced podocyte hypertrophy. In these studies, HG-induced podocyte hypertrophy was inhibited by the presence of a specific PTHrP neutralizing antibody. Moreover, PTHrP is able to upregulate the negative cell cycle regulatory protein p27Kip1, which plays a key role in diabetic cell hypertrophy by preventing activation of cyclin E activity and arresting the cell cycle later in G1 [71]. In addition, Rüster et al. reported that under HG conditions, AGEs induce podocyte cycle arrest and hypertrophy by stimulating the expression of p27Kip1 [72]. Romero et al. [71] found that the pharmacological blockade of PTH1R inhibited p27Kip1 upregulation induced by both HG and AngII. Taken together, these data suggest that PTHrP might mediate hypertrophic signaling, acting in an autocrine/intracrine fashion through the PTH1R receptor. Kim et al. [73] found that HG resulted in the activation of ERK1/2 and Akt/PKB and promoted podocyte hypertrophy, AngII can also increase ERK1/2 and Akt/PKB phosphorylation and cell hypertrophy in podocytes, and HG and AngII have a synergistic effect.

#### 3.1.4. IL-6/JAK2/STAT3 Signaling Pathway

**Figure 2:**
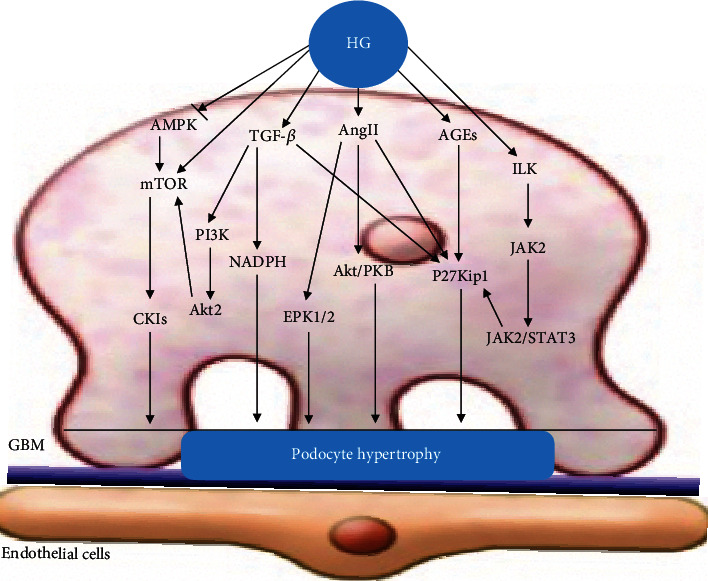
Podocyte hypertrophy. Elevated glucose upregulates TGF-*β*, mTOR, Ang II, AGEs, and integrin-linked kinase (ILK) pathways and activates the expression of p27Kip1, P38MAPK, Akt/PKB, and NADPH, which eventually leads to podocyte hypertrophy.

Interleukin-6 (IL-6) regulates cellular hypertrophy through the gp130/Janus kinase 2 (JAK2)/signal transducer and activator of transcription 3 (STAT3) pathway. Jo et al. [74] found that HG-stimulated podocytes produced and secreted IL-6, which activated the JAK2/STAT3 pathway via autocrine or paracrine mechanisms and participated in the process of cellular hypertrophy in vitro. This effect was attenuated by the addition of IL6NAbs to podocytes cultured under HG conditions, directly demonstrating that IL-6 was the cause of HG-induced podocyte hypertrophy. IL-6 might play a prominent role in the local activation of JAK2/STAT3 in podocyte hypertrophy under HG conditions. Local activation of the IL-6/JAK2/STAT3 pathway in podocytes could activate p21Cip and p27Kip1 expression. High expression of p27Kip1 may eventually lead to podocyte hypertrophy (Figure 2).

### 3.2. Podocyte EMT

#### 3.2.1. TGF-*β* Signaling Pathway

HG can increase the expression of TGF-*β*1. Numerous studies have shown that TGF-*β*1 is a master regulator governing the induction of EMT [75, 76]. Podocytes can be injured by hyperglycemia through the TGF-*β*/Smad classic pathway and multiple other pathways. Following the EMT process, the podocyte foot processes are effaced, which results in a loss of the slit diaphragm. The expression of nephrin, podocin, P-cadherin, and ZO-1 is downregulated, the actin cytoskeleton is rearranged, and the podocyte is no longer able to restrict urinary protein loss. This EMT process can finally cause podocyte-related DN. Activated TGF-*β* first integrates the TGF-*β* receptor type II (TbRII) and TGF-*β* receptor type I (TbRI) to form a ligand-receptor complex. This association results in the downstream phosphorylation and activation of Smad2 and Smad3. Phosphorylated Smad2/3 combines with Smad4 to form a Smad complex in the cytoplasmic domain, which gets translocated to the nucleus [77]. Then, activated TGF-*β* can lead to podocyte EMT. Stromal cell-derived factor-1*α* (SDF-1*α*), one of the substrates of DPP-4, can activate the protein kinase A pathway and subsequently inhibit its downstream effector, TGF-*β*1, which induces podocyte EMT. Chang et al. [78] found that SDF-1*α* plays an essential role in podocyte EMT inhibition.

#### 3.2.2. ILK Signaling Pathway

The ILK signaling pathway is also an important signaling pathway that mediates podocyte EMT. ILK, which binds the cytoplasmic domain of *β*1 integrin [79], is a serine-threonine kinase [80] and plays an important role in transmembrane signal transduction via integrins. Activated ILK causes phosphorylation of the downstream molecules Akt and GSK-3*β*. Phosphorylated Akt and GSK-3*β* can inhibit the phosphorylation of *β*-catenin, rendering high cytoplasmic concentrations of *β*-catenin. The cytoplasmic *β*-catenin will be transferred to the nucleus and binds to related transcription factors, which will cause the expression of Snail protein. Snail protein is an important protein that mediates podocyte EMT. Blockade of ILK activity with a highly selective small-molecule inhibitor reduced Snail induction and preserved podocyte phenotypes following TGF-beta1 or adriamycin stimulation. Kang et al. [81] found that ILK expression was induced in mouse podocytes by various harmful stimuli known to cause proteinuria, including TGF-*β*1, and high ambient glucose. Podocyte ILK was also found to be upregulated in human proteinuric glomerular diseases. Chen et al. [82] found that emodin ameliorated HG-induced EMT and subsequent podocyte dysfunction through nephrin upregulation, as well as desmin and ILK inhibition in vitro and in vivo. This reflects ILK-mediated HG-induced podocyte EMT. A previous study found that ILK upregulation and nephrin downregulation disrupted the balance of the ternary complex [83], which might be responsible for EMT. These results show that the upregulation of ILK is a convergent pathway leading to podocyte EMT.

#### 3.2.3. Wnt/*β*-Catenin Signaling Pathway

**Figure 3:**
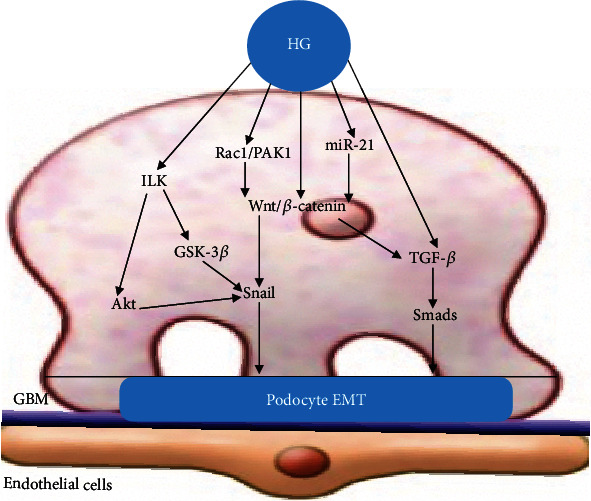
Podocyte EMT. HG activates the TGF-*β*, Notch, ILK, NF-*κ*B, and Wnt/*β*-catenin signaling pathways, which promotes the expression of Snail and eventually induces podocyte EMT.

The Wnt/*β*-catenin signaling pathway is also closely related to podocyte EMT [84]. Wnt/*β*-catenin is a highly conserved signaling pathway, and *β*-catenin is its core molecule. Li and Siragy [85] showed that in a HG environment, podocyte Wnt/*β*-catenin signaling is activated, and HG significantly decreased mRNA and protein expression of nephrin and increased mRNA and protein expressions of Wnt3a, *β*-catenin, and Snail. Snail, one of the downstream target genes of the Wnt-*β*-catenin signaling pathway, is an important transcription factor inducing podocyte EMT. Studies by Dai et al. [86] observed that Wnt1 was upregulated and *β*-catenin was activated in podocytes of human DN. Ectopic expression of either Wnt1 or stabilized *β*-catenin in vitro induced the transcription factor Snail and suppressed nephrin expression. Since Snail is the main transcription factor of EMT, so this study reflects that Wnt/*β*-catenin may promote podocyte transdifferentiation and phenotypic change mainly by promoting Snail expression. Another study showed that Rac1/PAK1 signaling contributed to HG-induced podocyte EMT via promoting *β*-catenin and Snail transcriptional activities [87]. In addition, miR-21 can upregulate the levels of TGF-*β*1 and P-Smad3 and reduce the level of Smad7 in the TGF-*β*1/Smads pathway by activating *β*-catenin, a key factor in the Wnt/*β*-catenin pathway, to promote podocyte dedifferentiation and podocyte EMT [88] (Figure 3).

### 3.3. Podocyte Detachment

#### 3.3.1. *α*3*β*1 Integrin

Podocytes and the GBM are closely connected and prevent the excretion of proteinuria via sustaining the glomerular filtration barrier. Podocyte detachment is closely related to the expression of adhesion molecules. Podocytes are anchored to the GBM by many molecules. Among them, integrin *α*3*β*1 is an important receptor that could tightly connect the podocyte to the GBM [89]. In podocytes cultured in vitro, TGF-*β*1 and mechanical stretching significantly reduce the expression of *α*3*β*1 integrin, reduce the adhesion function of podocytes, and promote podocyte detachment and apoptosis [90]. Kriz and Lemley [91] found that podocyte detachment depends on specific downstream effects: hypertension, ultrafiltration, and excessive glomerular growth, especially increased shear stress through the fissure membrane, and that mechanical forces are a key factor in the progression of glomerular disease to renal failure. Dai et al. [92] found that angiopoietin-like3 (Angptl3) is involved in podocyte detachment and apoptosis caused by puromycin, resulting in a large loss of podocytes. However, knockdown of Angptl3 by siRNA markedly ameliorated these injuries. Observed effects were partially correlated with the altered *α*3*β*1 integrin, ILK, and p53, rather than caspase-3. Chen et al. [93] found that the expression of *α*3*β*1 integrin on podocytes was suppressed in both humans and rats with diabetes, possibly due to the effects of hyperglycemia, and the suppression became more severe with the duration of diabetes. Susztak et al. [94] found that glucose-induced ROS production initiates podocyte depletion in vitro and in vivo. Further research showed that ROS can increase ILK expression in a dose-dependent manner, causing podocytes to detach [95]. Chen et al. [96] found that HG strongly inhibited the adhesion of podocytes to the BMC, which was accompanied by a reduction in *α*3*β*1 integrin mRNA and protein expression, as well as an increase in ILK activity and expression. Teixeira et al. [97] found that when glomerular ILK activity increases, *β*-catenin moves from the cell membrane into the cell, thus changing the cell phenotype and promoting the detachment of podocytes from the GBM. In addition, the overexpression of the ILK anchor protein repeat sequence can inhibit the binding of PINCH-1 to ILK, preventing the formation of the ternary complex, reducing the adhesion between podocytes and the GBM, and promoting apoptosis and exfoliation of podocytes [98]. It has been found that AngII stimulates podocytes to produce collagen IV, through TGF-*β* and VEGF signaling pathways, which leads to the accumulation of ECM, thickening of the GBM, and detachment of podocytes [57]. In addition, AngII can lead to oxidative stress, which in turn mediates the production of ROS and decreases the expression of *α*3*β*1 integrin.

#### 3.3.2. TGF-*β* Signaling Pathway

**Figure 4:**
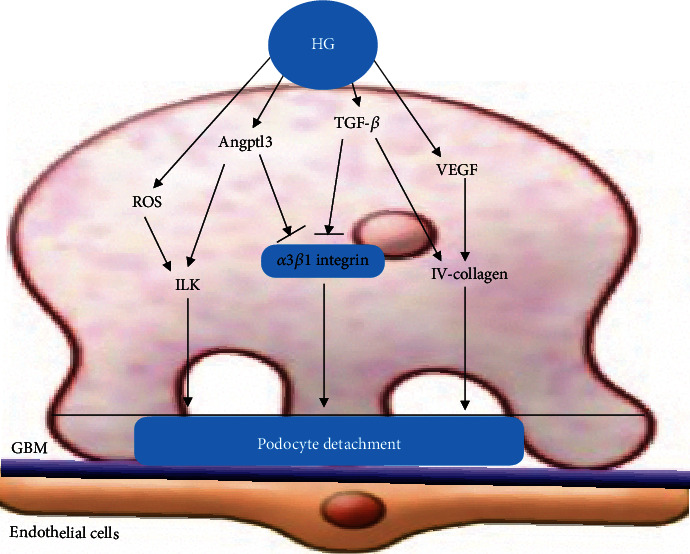
Podocyte detachment. HG can inhibit the expression of *α*3*β*1 integrin through Angptl3, and TGF-*β* pathways, thus promoting podocyte isolation. In addition, HG promotes podocyte detachment by upregulating ROS and VEGF pathways.

In the kidneys, TGF-*β*1 has been reported to be a strong regulator of the expression of integrins [99]. TGF-*β*1 has been demonstrated to suppress the expression of *α*3-integrin in the glomeruli of nephrotic rats [100]. HG can upregulate the expression of TGF-*β* receptor II; increase the sensitivity of podocytes to TGF-*β*; promote the paracrine effect in podocytes, mesangial cells, and glomerular endothelial cells; promote the secretion of TGF-*β*; and promote podocyte exfoliation. Dessapt et al. [90] found that downregulation of *α*3*β*1 integrin expression, by mechanical forces or TGF-*β*1, is sufficient to reduce podocyte adhesion (Figure 4).

### 3.4. Podocyte Apoptosis

#### 3.4.1. TGF-*β*1 Signaling Pathway

In the HG state, the TGF-*β*1 pathway is active and participates in podocyte apoptosis by mediating Smads, mTOR, and other signaling pathways. Das et al. [66] found that TGF-*β*1 selectively upregulates the transcription of Nox4 mRNA by Smad2/3, resulting in an elevated mitochondrial Nox4 protein level, oxidative stress, mitochondrial dysfunction, and apoptosis in podocytes. TGF-*β*1 induces podocyte apoptosis through the Erk-mediated mTORC1/Nox4 axis [101]. A study by Das et al. [101] found that TGF-*β*1 increases the translation of Nox4 through the Smad-ERK1/2-mTORC1 axis. Activation of this pathway plays a crucial role in ROS generation and mitochondrial dysfunction, leading to podocyte apoptosis. Gremlin plays an important role in regulating podocyte apoptosis. Overexpression of gremlin aggravates podocyte apoptosis. Gremlin is a developmental gene and is associated with DN [102]. Li et al. [103] found that HG induces increased expression of gremlin, which activates the TGF-*β*/Smad2/3 signaling pathway and aggravates podocyte apoptosis. Wang et al. [104] found that apoptosis-related proteins (Bcl-2 and Bax) are activated in the pathophysiology of DN. Overexpression of gremlin under HG conditions enhanced the expression of the TGF-*β* pathway, decreased the expression of the antiapoptotic gene Bcl-2, and increased the expression of the proapoptotic genes Bax and cleaved-caspase-3, causing podocyte apoptosis. TGF-*β*1 upregulated the expression of Cdk5 and p35 and Cdk5 kinase activity. HG increased the expression of Egr-1 via the TGF-*β*1-ERK1/2 pathway. Egr-1 is a member of a family of zinc-finger transactivators and is a known regulator of the p35 promoter. Inhibition of Cdk5 kinase activity alleviated podocyte apoptosis induced by HG or TGF-*β*1 [105]. Liu et al. [106] also found that HG stimulation increased the protein and mRNA expression of Cdk5 in a time-dependent manner in cultured mouse podocytes. The protein activator of Cdk5, p35, was also increased in a time-dependent manner by HG stimulation.

#### 3.4.2. AngII Signaling Pathway

HG promotes AngII expression and increases podocyte apoptosis. Liu et al. [107] found that AngII can increase podocyte apoptosis. AngII induces the relocalization and reduction of CD2AP via AT1R, which would cause podocyte apoptosis by the suppression of CD2AP/PI3-K signaling [108]. Treatment with AngII suppressed the viability and promoted the apoptosis of podocytes in a dose- and time-dependent manner. AngII decreased phosphor-Akt, phospho-p65 NF-*κ*B, nephrin, and podocin and increased caspase-9 expression, and podocyte apoptosis was promoted [109].

#### 3.4.3. AMPK Signaling Pathway

HG induces apoptosis in podocytes, inhibits AMPK activation, inactivates tuberin, and activates mTOR. HG also increases the levels of Nox4, Nox1, and NADPH oxidase activity [110]. Eid et al. [110] provided evidence that podocyte apoptosis in diabetic conditions is mediated by activation of the mTOR pathway through inactivation of AMPK. Lin et al. [111] found that HG stimulation negatively affects the mitochondria and reduces the sesn2 and p-AMPK levels, thereby promoting podocyte apoptosis. Cai et al. [112] found that grape seed proanthocyanidin extract, a strong antioxidant, prevents HG-induced mitochondrial dysfunction and apoptosis in podocytes via the AMPK-Sirt1-PGC-1*α* (Sirt1 silent information regulator T1) pathway. This study reflects AMPK-mediated podocyte apoptosis. Eid et al. [113] found that inactivation of AMPK by HG upregulated the expression and phosphorylation of p53, and p53 acted downstream of Nox4. To investigate the mechanism of podocyte apoptosis in vivo, they used OVE26 mice, a model of type 1 diabetes. Glomeruli isolated from these mice showed decreased phosphorylation of AMPK and enhanced expression of Nox4 and p53. Their results uncover a novel function of AMPK that integrates metabolic input to Nox4 and provides new insight for the activation of p53 to induce podocyte apoptosis.

#### 3.4.4. ROS Signaling Pathway

In DN, excessive ROS production induced by HG decreases the number of podocytes. Related studies have confirmed [114] that mitochondria are considered to be an important source of intracellular ROS and participate in the endogenous apoptosis pathway. Previous studies have demonstrated that ROS was increased in podocytes [115] and that HG-induced ROS increase promotes podocyte apoptosis in DN [94]. Further research found that ROS can activate the P38 MAPK pathway, which may be an important pathological mechanism of podocyte apoptosis induced by oxidative stress [116]. Susztak et al. [94] found that HG rapidly stimulated the generation of intracellular ROS through NADPH oxidase and mitochondrial pathways and led to the activation of proapoptotic p38 MAPK and caspase-3 and to the apoptosis of conditionally immortalized podocytes in vitro. Among the members of the NADPH oxidase family, Nox4 is the key enzyme in ROS production and podocyte apoptosis induced by oxidative stress in DN [117, 118]. Liu et al. found that [119] metadherin is a potent modulator of podocyte apoptosis and that it represents the target of miR-30s, facilitating podocyte apoptosis through the activation of a HG-induced p38 MAPK-dependent pathway. In addition, Wang et al. [120] indicated that inhibiting mitochondrial oxidative damage and the release of cytochrome C can eliminate ROS, thereby preventing the transmission of podocyte damage and apoptosis signals.

#### 3.4.5. Endoplasmic Reticulum Stress (ERS) Signaling Pathway

Activated ERS plays an important role in podocyte apoptosis in HG environments. HG can initiate ERS through a variety of ways. AGE products are important in the pathogenesis of DN. AGEs can upregulate the expression of glucose-regulated protein 78 in podocytes, induce ERS, and eventually lead to podocyte apoptosis in a dose- and time-dependent manner [121]. Lei et al. [122] found that the activated mTOR by ERK1/2 results in energy consumption, which in turn leads to ERS signaling and triggers apoptosis in HG-treated podocytes. Cao et al. [123] found that ER stress inhibitors ursodeoxycholic acid (UDCA) or 4-phenylbutyrate (4-PBA) prevented hyperglycemia-induced or HG-induced apoptosis in podocytes in vivo and in vitro via the inhibition of caspase-3 and caspase-12 activation. In addition, Shen et al. [124] found that TUG1 was highly expressed in cells following treatment with HG, and PGC-1*α* and cleaved-caspase-3 levels were much lower, while CHOP levels were much higher. Furthermore, CHOP inhibited PGC-1*α* expression. TUG1 negatively regulated CHOP expression and positively regulated PGC-1*α* expression. Their study suggested that the long noncoding RNA (lncRNA) TUG1 influenced podocyte apoptosis via mediating the ERS-CHOP-PGC-1*α* signaling pathway in HG-induced DN.

#### 3.4.6. Other Related Signaling Pathway

**Figure 5:**
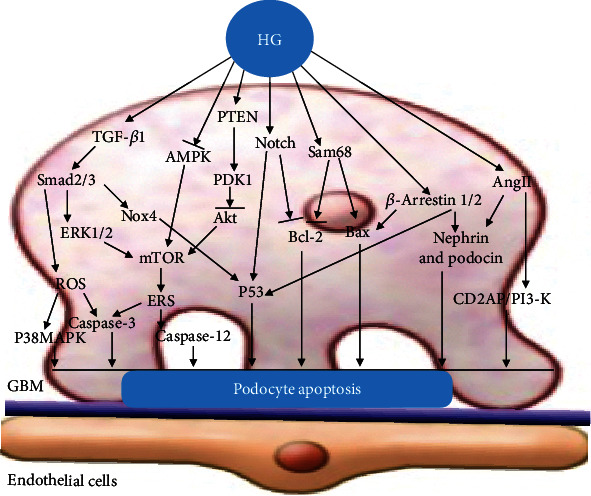
Podocyte apoptosis. HG can stimulate the upregulation of TGF-*β*, mTOR, Notch, AngII, Sam68, ROS, and ERS signal pathways, inhibit the activity of AMPK and expression of Bcl-2, and activate the apoptosis pathways of p53, Bax, caspase-3, P38MAPK, CD2AP/PI3-K, and caspase-12, to induce podocyte apoptosis.

Under normal circumstances, proapoptotic and antiapoptotic signaling pathways coexist to maintain the body's homeostasis. The podocyte-associated proteins nephrin and podocin have antiapoptotic signal transduction properties. *β*-Arrestin 1/2 expression levels of podocytes were found to be upregulated in HG-induced podocytes, and *β*-arrestin 1/2 overexpression inhibited the expression of nephrin and podocin proteins. Upregulated *β*-arrestin 1/2 promoted podocyte apoptosis and the p53 pathway by increasing Bax, cleaved-caspase-3, and p-p53 levels in HG-induced podocytes [125]. High expression of PVT1 or low expression of FOXA1 can downregulate the expression of synaptophysin and podocyte protein, decrease the expression of Bcl-2, and increase the expression of Bax and cleaved-caspase-3, thus promoting podocyte apoptosis [126]. Chen et al. [127] found that Sam68 was upregulated in a time- and dose-dependent manner in in vitro HG-treated podocytes. Furthermore, HG increased Bax and decreased Bcl-2 protein expression in cultured podocytes, and this effect was blocked by Sam68 knockdown. Their results showed that Sam68 mediated HG-induced podocyte apoptosis, probably through the Bax/Bcl-2 signaling pathway. Gao et al. [128] found that HG upregulated the Notch pathway in podocytes, which was accompanied by the alteration of Bcl-2 and p53 pathways, subsequently leading to podocyte apoptosis. In addition, Wang et al. [120] found that SS-31 prevented oxidative stress and mitochondria-dependent apoptosis signaling by hypochlorite-modified albumin (HOCl-alb) in vivo and in vitro, as evidenced by the release of cytochrome c, the binding of apoptosis activated factor-1 (Apaf-1) and caspase-9, and the activation of caspases. These data suggest that SS-31 may prevent podocyte apoptosis, exerting renal protection in diabetes mellitus, probably through an apoptosis-related signaling pathway involving oxidative stress. A study by Feng et al. [129] found that mitochondrial pyruvate carrier 2 may mediate mitochondrial dysfunction in HG-treated podocytes, ultimately leading to cell apoptosis. High D-glucose (HDG) significantly increased PLK2 expression in mouse podocytes. Suppressing PLK2 attenuated HDG-induced apoptosis and inflammatory responses both in vitro and in vivo [130]. Bai et al. [131] found that VEGF-A inhibition ameliorates podocyte apoptosis by regulating activator protein 1 (AP-1) and Bcl-2 signaling. AP-1 is a direct target of VEGF-A and a novel component of podocyte apoptosis. Recent studies have found that HG can promote podocyte apoptosis through the PTEN-PDK1-Akt-mTOR pathway [132] (Figure 5).

## 4. Conclusion

**Figure 6:**
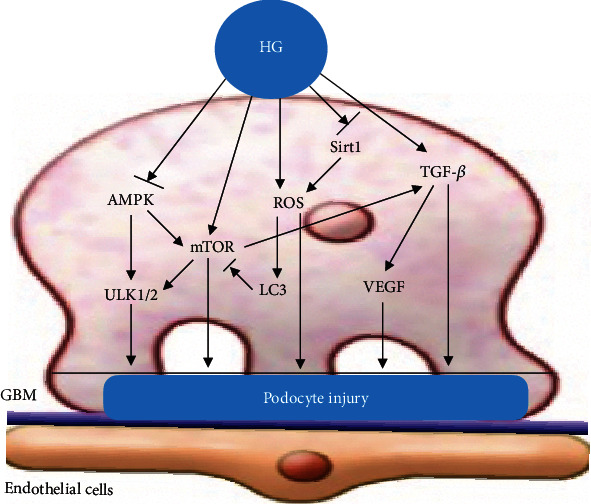
The function of podocyte injury is closely related to morphological changes. HG inhibits podocyte autophagy and promotes podocyte hypertrophy by activating the mTOR signaling pathway. HG inhibits podocyte autophagy and promotes podocyte apoptosis by inhibiting AMPK and activating ROS signaling.

Podocyte injury is a key factor in the occurrence and development of DN. Most studies have shown that the pathological mechanism of podocyte injury mainly includes four morphological changes: podocyte hypertrophy, podocyte EMT, podocyte detachment, and podocyte apoptosis, as well as functional changes in podocyte autophagy. There is a close relationship between the function and morphology of podocyte injury. For example, HG conditions activate mTOR signaling to inhibit podocyte autophagy and promote podocyte hypertrophy and podocyte EMT. Alternatively, HG conditions inhibit AMPK and generate ROS to inhibit podocyte autophagy and promote podocyte apoptosis (Figure 6). In the early lesions of DN, abnormal podocyte function is particularly prominent, including the downregulation of key structural molecular proteins in the podocyte fissure membrane, or protein binding disorders of adjacent structural molecules. With the deepening of research, more and more pathways concerning podocyte injury have been revealed. However, DN is a serious global health problem, and the potential pathological mechanisms of podocyte morphological and functional damage require further investigation.
